# Supplementary material for: Systematic review and theoretical comparison of children’s outcomes in post-separation living arrangements
Source: PLoS One. 2023 Jun 30;18(6):e0288112. doi: 10.1371/journal.pone.0288112 (PMC10313020; doi:10.1371/journal.pone.0288112)
Supplement: Attachment — Submitted filename: manuscript review PlosOne.docx [file pone.0288112.s002.docx]

Title: Systematic Review and Theoretical Comparison of Children’s Outcomes in Post-Separation Living Arrangements

The authors have conducted a thorough review of literature with relevant and recent studies. This work will be helpful to researchers, professionals, and more importantly policymakers working in the field of union dissolution. The conclusion is also very clear from the systematic review and the limitations well highlighted. My concerns about organization and format of certain elements of the manuscript that the authors may deliberate upon, are provided below.

1. Parentheses should not be used for explaining the term or phrase.

Page 3: 1^st^ paragraph has shared custody outside the parenthesis but what exactly is it, is mentioned inside the parenthesis. The same has been repeated for SPC and nuclear families. It has been repeated with other newly introduced terms and phrases as well.

Mentioning the abbreviations LPC and SPC inside the parenthesis is correct.

On page 14: under the heading Screening and Selection the examples of types of documents are mentioned inside the bracket (e.g., editorials, literature reviews…). This should be avoided as per APA. The authors are suggested to check section 6.8 on Parentheses in APA Publication Manual (7^th^ ed.).

2. Page 3: 2^nd^ paragraph for example appears after the colon and then for e.g. appears inside the parentheses again. Repetition can be avoided after colon. The sentences can be reframed as follows:

Some studies have tried to distill factors that may explain the differences in children’s outcomes across living arrangements. Some of these factors are: socioeconomic status (e.g., Amato et al., 2011; Bergström et al., 2014; 2015; 2019; Fransson et al., 2018),…

3. Page 4: Several of these reviews have focused primarily on the differences between SPC and LPC families showing that, overall, children tend to do better in SPC than in LPC families (Baude et al., 2016; 2019; Nielsen, 2014; 2018).

For e.g., needs to be added inside the parentheses before Baude et al., 2016 in the above mentioned sentence if the conclusion that several of these reviews have focused primarily on the differences between SPC and LPC families showing that, overall, children tend to do better in SPC than in LPC families has been drawn by the authors and not the researchers mentioned in the parentheses. It seems that the conclusion was drawn by referring to their works and not by them. But the way it is currently written, it seems that the conclusion was drawn by (Baude et al., 2016; 2019; Nielsen, 2014; 2018).

This has been repeated on page 8 as well as in other places in the manuscript where the conclusion was drawn by the authors but it seems like the researchers cited have mentioned the same.

4. Page 4: The authors may consider placing the definition of nuclear families with other definitions of LPC and SPC in preceding paragraphs.
5. Page 5: 2^nd^ line, Jensen and Sanner – please mention the year in parenthesis.

Page 5: The portion on social capital perspective is in parentheses and there is another citation within it. The authors are suggested to check section 6.8 on Parentheses in APA Publication Manual (7^th^ ed.) to check where the parentheses can be used and if the use of brackets is needed within the parentheses then which type of bracket is used.

6. Page 7: The last four lines of the paragraph may be moved to some other place; the text would read better if the description of explanatory variables are mentioned before introducing their categories.
7. The four main hypotheses are written in bold within the paragraph. As per APA bold is usually used for headings and certain elements in tables and figures. The authors are suggested to check the guidelines for the same.
8. The main hypothesis is in bold but the other supporting/associated ones are in italics. However, this format has not been followed for context hypothesis within selection hypothesis on page 8. For better presentation, please follow a uniform format for all four hypotheses.
9. Page 9: The escape from stress theory sentence is very lengthy. Consider breaking it into two so that the meaning is effectively delivered.
10. Page 11: The example may be placed in the section above (versus ending the paragraph with it). “Thus, we expect that economic and/or relationship variables will explain at least some of the differences in between living arrangements” may be used as a concluding statement.
11. Page 12: Please provide some elaboration on the PICO components with reference to your study.
12. Page 13: It has been mentioned that the following population and contexts were included: Western European countries, North American countries, Australia, and New Zealand where divorce and shared physical custody have become increasingly common. The authors are suggested to be support this with existing research.
13. Page 15: Under the heading- Comparison of the Empirical Literature with the Theoretical Hypotheses- Most studies supported the fewer resources hypothesis (51.3%) suggesting that children from divorced or separated families have fewer resources than nuclear families with children living in LPC arrangements having the least access to relational and economic resources- is not clear. What are nuclear families with children living in LPC arrangements? How is it a nuclear family if it is an LPC arrangement? – please clarify.
14. Page 17: Methodologies – the heading not clear. What is the justification behind placing it after the Results and before the Discussion.
15. Page 20: 2^nd^ paragraph: The sentence starts with “Some theories and research findings suggest that the introduction of stepparents…” - and studies are cited in parentheses but it concludes with – but this was not supported by literature. This first part of the sentence mentions certain studies specifically; then how do the authors claim that literature does not support the statement? Are there contradicting studies in the area? If so, then please state this categorically.
16. Page 31: Capitalise R in roles as the word appears after colon in the following reference-

*Dissing, A. S., Dich, N., Andersen, A. N., Lund, R., & Rod, N. H. (2017). Parental break-ups and stress: roles of age & family structure in 44 509 pre-adolescent children. European Journal of Public Health, 27(5), 829–834. https://doi.org/10.1093/eurpub/ckx040
