# Supplementary material for: Systematic review and theoretical comparison of children’s outcomes in post-separation living arrangements
Source: PLoS One. 2023 Jun 30;18(6):e0288112. doi: 10.1371/journal.pone.0288112 (PMC10313020; doi:10.1371/journal.pone.0288112)
Supplement: Attachment — Submitted filename: 230525PLOSresponse_to_reviewers.docx [file pone.0288112.s003.docx]

May 25, 2022

Dear Dr Banerjee,

We appreciate the work of both you and the dedicated reviewers in providing valuable feedback on our manuscript. We have addressed the reviewers’ comments in our revised manuscript entitled “Systematic review and theoretical comparison of children’s outcomes in post-separation living arrangements”. It is attached for further consideration.

We have responded to the reviewer requests (indicated in **bold** font) point-by-point below in this cover letter and quoted the changes in the manuscript. We have also included a manuscript with track changes. This study has not been published previously and is not currently under consideration for publication elsewhere. We agree not to submit the full report to another journal.

**Editorial comments:**

**1. Please ensure that your manuscript meets PLOS ONE's style requirements, including those for file naming. The PLOS ONE style templates can be found at** 
[**https://journals.plos.org/plosone/s/file?id=wjVg/PLOSOne_formatting_sample_main_body.pdf**](https://journals.plos.org/plosone/s/file?id=wjVg/PLOSOne_formatting_sample_main_body.pdf) **and** 
[**https://journals.plos.org/plosone/s/file?id=ba62/PLOSOne_formatting_sample_title_authors_affiliations.pdf**](https://journals.plos.org/plosone/s/file?id=ba62/PLOSOne_formatting_sample_title_authors_affiliations.pdf)

We have reformatted the article based on the PLOS ONE style requirements.

**2. Thank you for submitting the above manuscript to PLOS ONE. During our internal evaluation of the manuscript, we found the PROSPERO Registration number for the SRMA is missing. We would also need to know which authors took part in the literature review of the studies in the SRMA. Please Resubmit with the required. Thank you for your attention. We look forward to hearing from you.**

We did not register the systematic review on PROSPERO. Instead, the protocol was published on the Open Science Framework: <https://osf.io/9w5rp/>. We have added this statement in the methods: *“The systematic review was not registered on PROSPERO but all the material pertaining to the review including the protocol as well as all supplemental files can be found on the Open Science Framework:* [*https://osf.io/9w5rp/*](https://osf.io/9w5rp/)*.”*

We have added a clarification on who took part in the literature review in the paper: *“DCM, CLC, and LMV conducted the initial selection based on titles and abstracts, DCM and LMV read all full texts for the final selection of articles. “*

**3. Thank you for stating the following financial disclosure: 
"This study received funding “Seeds of LIVES” from the Interdisciplinary Research Centre LIVES, University of Lausanne, Switzerland paid to JD."
  
Please state what role the funders took in the study.  If the funders had no role, please state: "The funders had no role in study design, data collection and analysis, decision to publish, or preparation of the manuscript." 
If this statement is not correct you must amend it as needed. 
Please include this amended Role of Funder statement in your cover letter; we will change the online submission form on your behalf.**

This study received funding “Seeds of LIVES” from the Interdisciplinary Research Centre LIVES, University of Lausanne, Switzerland paid to JD. The funders had no role in study design, data collection and analysis, decision to publish, or preparation of the manuscript.

**4. Please note that in order to use the direct billing option the corresponding author must be affiliated with the chosen institute. Please either amend your manuscript to change the affiliation or corresponding author, or email us at** [**plosone@plos.org**](mailto:plosone@plos.org) **with a request to remove this option.**

The corresponding author is affiliated with the chosen institute.

**5. Please ensure that you include a title page within your main document. You should list all authors and all affiliations as per our author instructions and clearly indicate the corresponding author.**

We have now included the title page with the correct formatting within the main document.

**5. Review Comments to the Author**

**Reviewer #1: Section1
Overall, this section provides a comprehensive overview of the main theoretical hypotheses and related theories explaining differences in children's outcomes across living arrangements. However, some suggestions for improvements and comments are as follows:**

We thank the reviewer for their kind comments and very helpful suggestions.

**Consider reorganizing the section to enhance clarity and flow. For example, you could introduce each main hypothesis and then provide a brief summary of the related theories before delving into details. This will help readers understand the connections between the hypotheses and their supporting theories more easily.**

We thank the reviewer for the suggestion, and we have reorganized this section as suggested. For example, under “selection hypothesis”, we now write: *“The selection hypotheses [24–28] states that children in separated and non-separated families differ because of the family characteristics that precede separation. The hypothesis suggests that it is not the family living arrangement after separation that makes children in these families more vulnerable but rather that the separation itself is a consequence of their already existing higher vulnerability. Previous literature suggests that non-nuclear families, already before separation, are less wealthy and less educated, tend to display lower quality of parent-child relationship, greater levels of conflict, lower co-parenting, and investment in children [24–28]. Furthermore, income, conflict, parent-child relationships, and quality of parenting skills are the factors most frequently proffered as the reasons why SPC children have better outcomes than LPC children [6,29]. Based on the selection hypothesis, we expect no significant differences across the living arrangements after accounting for selection variables in the analyses.*

*In addition to the selection hypothesis, the context hypothesis (based on the bioecological model) also suggests that it is not the living arrangements themselves that affect children’s outcomes, but it is the context within which the children live. The context hypothesis suggests that it is the overall context (e.g., conflict between family members, social environment) that affects children’s outcomes rather than living arrangements alone. Thus, it also assumes that differences in living arrangements are due to selection. Therefore, these two hypotheses are combined in Table 1 under selection hypothesis. Based on the context hypothesis, other possible explanatory variables include relationships (e.g., parent-child relationship quality, conflict between parents), parent’s well-being, child variables, and economic situation as these form a part of the bioecological system.”*

**For each hypothesis, include a brief explanation of how the explanatory variables are relevant to the hypothesis. This will help readers understand the potential mechanisms through which living arrangements might influence children's outcomes.**

We have now provided a brief explanation of the explanatory variables throughout.

For selection hypothesis: *“Based on the selection hypothesis, we expect no significant differences across the living arrangements after accounting for selection variables in the analyses. Based on the context hypothesis, other possible explanatory variables include relationships (e.g., parent-child relationship quality, conflict between parents), parent’s well-being, child variables, and economic situation as these form a part of the bioecological system.”*

For instability hypothesis: *The expected explanatory variables based on the instability hypothesis for the differences between living arrangements would include the number of transitions as well as having a stepparent. Additionally, the escape from stress theory would also suggest that the differences may be due to a single parent’s poorer well-being or poorer parent-child relationship.*

We had already included this for the fewer resources hypothesis: *“Thus, we expect that economic and/or relationship variables will explain at least some of the differences between living arrangements.”*

Stressful mobility hypothesis: *“First, the stressful mobility hypothesis assumes that having to move between households (potential explanatory variable) can be stressful for children with more frequent moves adding to the stress [41, 42]”* and *“Potential explanatory variables based on this theory would include the number of moves between homes and relationship quality between the parents as well as between parents and their children.”*

**The section on the Stressful Mobility Hypothesis could be further developed by discussing how the frequency of movement between households may influence the level of stress experienced by children. Additionally, consider providing more information on how the age of the child and the level of conflict between parents might moderate the impact of mobility on children's outcomes.**

We have added this information: *“First, the stressful mobility hypothesis assumes that having to move between households (potential explanatory variable) can be stressful for children with more frequent moves adding to the stress [41,42], and that the level of stress due to mobility can vary depending on the age of the child and the level of conflict between parents [36]. In one study [36], older children reported higher stress when moving between households compared to younger children. This may be, for example, because the moves cause disruption in their friendships or out-of-school activities. They also showed that children in higher-conflict families showed higher levels of stress in SPC arrangements compared to children in low-conflict families."*

**To strengthen the arguments, consider citing more recent literature to support each hypothesis and related theories, especially those that have emerged since your knowledge cutoff in 2021.**

We have included papers in the systematic review until December 2022.  **Be mindful of the terminology used throughout the section. For example, instead of referring to "nuclear" and "non-nuclear" families, consider using more precise and inclusive terms, such as "intact" and "non-intact" families, respectively.**

We chose to use the term “nuclear families” because a previous reviewer asked us to change from “intact families” to “nuclear families” stating that “nuclear families” has a less negative connotation because non-intact may refer to a fragmented, broken family. Both terms are used in the literature and have both neutral and negative connotations associated with them. In the end, we chose to keep the term “nuclear family” instead of “intact family”.

**In the conclusion of this section, consider summarizing the main hypotheses and their implications for the systematic review. This will help readers understand the relevance of the reviewed theories to the overall research question and how they can inform the interpretation of the review findings.**

We have added a conclusion to this section: *“In summary, these four main hypotheses (selection hypothesis, instability hypothesis, fewer resources hypothesis, and stressful mobility hypothesis) derived from several major theories within psychology and sociology suggest differing outcomes across living arrangements. They also suggest several possible explanatory variables that may explain the differences in children’s outcomes across living arrangements. In the systematic review presented in the section below, we aim to compare the empirical findings against these hypotheses.”*

**Section 2: The Systematic Review
Methods
Overall, this section provides a detailed description of the systematic review process, including the methods, inclusion and exclusion criteria, search strategy, screening and selection, and data extraction. However, there are a few suggestions for improvements and comments to enhance clarity and organization:**

We would like to thank the reviewer for their acknowledgement of the detailed information we have provided in this section.

**When listing the inclusion and exclusion criteria, use bullet points to make it easier for the reader to quickly understand the criteria. For example:

Publications must focus on divorce or separation of cohabiting or married couples.
They must include outcomes for children and young people aged between 0-18 years old.
Studies should include comparisons with intact families, SPC, and LPC.**

We have changed the section now to include numbered bullet points instead of a paragraph.

**In the "Inclusion and Exclusion Criteria" section, consider using more inclusive terminology, as previously mentioned. For example, replace "nuclear families" with "intact families."**

We chose to use the term “nuclear families” because a previous reviewer asked us to change from “intact families” to “nuclear families” stating that “nuclear families” has a less negative connotation. Both terms are used in the literature and have both neutral and negative connotations associated with them. In the end, we chose to keep the term “nuclear family” instead of “intact family”.

**In the "Search Strategy" section, you mentioned four electronic bibliographic databases, but only listed three (Scopus, Web of Science Core Collection, and APA PsycInfo). Please correct this by either removing the mention of the fourth database or adding the fourth database to the list.**

We’ve changed the word “four” with “three”.

**In the "Screening and Selection" section, consider adding a sentence or two explaining how discrepancies or uncertainties in the screening and selection process were resolved, such as through discussion among the research team or consulting with an expert in the field.**

We had already mentioned this in the section: “When unclear, we consulted and resolved the selection with the principal researchers.”

**In the "Data Extraction" section, the reference to Nielsen (2018) should be formatted consistently with other references throughout the text. Check that all in-text citations and references are formatted according to the chosen citation style.**

PLOS ONE doesn’t use APA citation formatting so for the revised submission we have changed the citations to conform with PLOS ONE guidelines. Therefore, this comment is no longer relevant.

**Consider including a brief summary of the main findings of the systematic review at the end of this section. This will help readers understand the main outcomes and trends identified in the review.**

We believe the main findings should be presented in the results section (as per PRISMA guidelines) and have thus not included the main findings in the methods.

**Finally, carefully proofread the entire section for any grammatical, punctuation, or formatting errors to ensure clarity and readability. For example, you have a few instances of extra spaces between words that should be removed**

We have proofread this section.

**Results/Methodologies
This section presents the results of the systematic review and provides a comparison of the empirical literature with the theoretical hypotheses. To improve clarity and readability, consider the following suggestions:

In the first paragraph, rephrase the sentence "Thus, in the table we split the studies that made an explicit comparison and those that did not under the hypothesis" for clarity. Consider: "In the table, we have separated the studies that made explicit comparisons from those that did not, under each hypothesis."**

We have changed the sentence to the rewording suggested by the reviewer.

**In the "Do the Results Differ Based on Which Outcomes Were Examined in the Studies?" section, consider breaking the lengthy paragraph into smaller paragraphs to improve readability.**

We have split the paragraph into three smaller paragraphs.

**In the "Are the Results Better Explained by Certain Explanatory Variables?" section, consider using bullet points when listing the types of explanatory variables. For example:

Selection (parent demographic variables; n = 14)
Relationships (e.g., parenting, parents’ relationship quality; n = 15)
Economic (e.g., education, occupation, income; n = 17)
Child (e.g., age, gender; n = 20)
Having a stepparent (n = 12)**

We have changed this section to bullet points.

**In the same section, rephrase the sentence "Most of the time the inclusion of explanatory variables did not change the significance of the results" to clarify the meaning. Consider: "In most cases, the inclusion of explanatory variables did not alter the significance of the results."**

We have changed the sentence to the rewording suggested by the reviewer.

**In the "Methodologies" section, add a subheading to separate it from the previous section, and consider breaking the lengthy paragraph into smaller paragraphs for better readability.**

We have changed this section to “methodological findings”. This section is still part of the results with its own subheading. We have split the paragraph into two. **Be consistent with the use of percentages in the text. For example, use "5.1% of the studies" instead of "two of the 39 (5.1%) studies" and "41.0% of the studies" instead of "16 (41.0%) studies."**

We have checked the text for consistency with the use of percentages throughout the results section. **Carefully proofread the entire section for any grammatical, punctuation, or formatting errors to ensure clarity and readability. For example, you have a few instances of extra spaces between words that should be removed.**

We have proofread this section.

**Discussion/ Implications for Theory, Research, and Practice

In this section, the authors present a systematic review on the effects of different family living arrangements on children's outcomes. They also discuss the implications of the findings for theory, research, and practice. The following suggestions may help improve clarity and readability:

In the first paragraph, consider breaking down the long sentences into shorter ones for better readability. For example, separate the discussion of the two main conclusions and further divide the discussion of each conclusion into multiple sentences.**

We have split the long sentences into multiple sentences.

**In the second paragraph, it would be helpful to clarify which literature supports the stressful mobility hypothesis, as the text states it is not supported by the literature, yet cites a review by Nielsen (2018) that appears to support it.**

Nielsen (2018) article finds that children in SPC arrangements have better outcomes than children in LPC arrangements thus not supporting the stressful mobility hypothesis. There is no literature included in the systematic review which supported the stressful mobility hypothesis.

**In the paragraph discussing the implications for theory, research, and practice, consider reorganizing the content to follow a clear structure. Start by discussing the implications for theory, followed by research implications, and finally practice implications. This will make it easier for readers to understand the main points of each section.**

This section is already organized as the reviewer suggests. We first discuss the implications for theory, then discuss the importance of making a theoretical background clear in research (research implication), and finally implications for social policies and legal procedures. To take into account the reviewer’s comment, we have clarified in the second paragraph that we are talking about implications for researchers by adding “in research” into the first sentence: *“Furthermore, the potential effects of living arrangements on children’s outcomes may be conceptualized differently depending on the theoretical perspective adopted in research, yet most of the studies were atheoretical.”*

**In the discussion of practice implications, consider adding more specific examples of policies or interventions that could be used to support high-risk families and promote gender equality in the home and childcare.**

We have elaborated on the implications to policy and interventions on pages 34-35: *“Thus, supporting high risk families should be the first focus of policies given a stable nuclear family is likely to provide the most supportive environment for children to grow. This may include, for example, preventive measures such as family and gender policies which facilitate a more equal share of household and childcare tasks (sustainable parental leave policies, encouraging gender-equal pay at work).* *In cases where a couple chooses to end their relationship, the results suggests that policies should support families to choose and maintain an SPC arrangement and in many cases, it is possible to achieve comparable outcomes to nuclear families. This could be achieved, for example, by discouraging long and conflictual divorce proceedings and instead encouraging shorter and consensual separation procedures which center the best needs of the child(ren).*

*In addition to policy changes, providing interventions for couples to address their coparenting relationship can help improve children’s outcomes. For example, parents' participation in a co-parenting program has been shown to improve overall family well-being [82]. In these programs, parents receive psychoeducation on the importance of their relationship quality for their child, practice communication skills, and are supported in developing co-parenting plans. These programs are designed for parents both before and after separation or divorce [83].”*

**The last paragraph could benefit from a stronger concluding statement that summarizes the main findings and implications of the review. Emphasize the importance of using research-based evidence in shaping social policies and legal procedures related to family living arrangements and children's well-being.**

We have reworded the concluding paragraph, which now states: *“Several main messages can be drawn from the existing literature on the association between living arrangements and children’s well-being: (1) children in nuclear families do better than children in various post-separation living arrangements; however, different explanatory variables change the association between living arrangements and children’s outcomes in inconsistent ways; (2) children in SPC arrangements are consistently found to do better than children in LPC arrangements and to do equally well compared to children in nuclear families; (3) children in stepfamilies show equal outcomes compared to LPC. In conclusion, the findings from the existing literature provide research-based evidence to shape social policies and legal procedures related to family living arrangements and children well-being.”*

**Additionally, throughout the section, consider checking the citation formatting to ensure consistency. Some citations have multiple years, while others have a single year with multiple letters (e.g., 2019; 2014; 2019 vs. 2021a; 2021c). Make sure the citation style is consistent throughout the text.**

PLOS ONE doesn’t use APA citation formatting so for the revised submission we have changed the citations to conform with PLOS ONE guidelines. Therefore, this comment is no longer relevant.

**Reviewer #2: The authors have conducted a thorough review of literature with relevant and recent studies. This work will be helpful to researchers, professionals, and more importantly policymakers working in the field of union dissolution. The conclusion is also very clear from the systematic review and the limitations well highlighted. My concerns about organization and format of certain elements of the manuscript that the authors may deliberate upon, are provided.**

We thank the reviewer for their kind comments and have included their comments and our responses below.

1. **Parentheses should not be used for explaining the term or phrase.**

**Page 3: 1^st^ paragraph has shared custody outside the parenthesis but what exactly is it, is mentioned inside the parenthesis. The same has been repeated for SPC and nuclear families. It has been repeated with other newly introduced terms and phrases as well.**

**Mentioning the abbreviations LPC and SPC inside the parenthesis is correct.**

**On page 14: under the heading Screening and Selection the examples of types of documents are mentioned inside the bracket (e.g., editorials, literature reviews…). This should be avoided as per APA. The authors are suggested to check section 6.8 on Parentheses in APA Publication Manual (7^th^ ed.).**

We have removed as many parentheses as possible. However, there were some parts of the text where we felt that in the end the parentheses were the best option to provide these explanations, or additional information, to the terms or phrases.

2. **Page 3: 2^nd^ paragraph for example appears after the colon and then for e.g. appears inside the parentheses again. Repetition can be avoided after colon. The sentences can be reframed as follows:**

**Some studies have tried to distill factors that may explain the differences in children’s outcomes across living arrangements. Some of these factors are: socioeconomic status (e.g., Amato et al., 2011; Bergström et al., 2014; 2015; 2019; Fransson et al., 2018),…**

We have changed this phrase as the reviewer suggested. It now reads: *“Several studies have tried to distil factors that may explain the differences in children’s outcomes across living arrangements. Some of these factors include: socioeconomic status [4,7–9]…”*

3. **Page 4: Several of these reviews have focused primarily on the differences between SPC and LPC families showing that, overall, children tend to do better in SPC than in LPC families (Baude et al., 2016; 2019; Nielsen, 2014; 2018).**

**For e.g., needs to be added inside the parentheses before Baude et al., 2016 in the above mentioned sentence if the conclusion that several of these reviews have focused primarily on the differences between SPC and LPC families showing that, overall, children tend to do better in SPC than in LPC families has been drawn by the authors and not the researchers mentioned in the parentheses. It seems that the conclusion was drawn by referring to their works and not by them. But the way it is currently written, it seems that the conclusion was drawn by (Baude et al., 2016; 2019; Nielsen, 2014; 2018).**

**This has been repeated on page 8 as well as in other places in the manuscript where the conclusion was drawn by the authors but it seems like the researchers cited have mentioned the same.**

The conclusion of these articles is that children tend to do better in SPC than in LPC families. Due to the Vancouver citation format, we have not added for e.g., in the text.

4. **Page 4: The authors may consider placing the definition of nuclear families with other definitions of LPC and SPC in preceding paragraphs.**

We have not done so as we do not introduce the concept of nuclear families within the introductory paragraph given the review by Nielsen only focuses on SPC vs. LPC arrangements following divorce.

5. **Page 5: 2^nd^ line, Jensen and Sanner – please mention the year in parenthesis.**

**Page 5: The portion on social capital perspective is in parentheses and there is another citation within it. The authors are suggested to check section 6.8 on Parentheses in APA Publication Manual (7^th^ ed.) to check where the parentheses can be used and if the use of brackets is needed within the parentheses then which type of bracket is used.**

PLOS ONE doesn’t use APA citation formatting so for the revised submission we have changed the citations to conform with PLOS ONE guidelines. Therefore, this comment is no longer relevant.

6. **Page 7: The last four lines of the paragraph may be moved to some other place; the text would read better if the description of explanatory variables are mentioned before introducing their categories.**

We have moved this part to just above the introduction of the explanatory variables.

7. **The four main hypotheses are written in bold within the paragraph. As per APA bold is usually used for headings and certain elements in tables and figures. The authors are suggested to check the guidelines for the same.**

We have removed the bold formatting within these paragraphs.

8. **The main hypothesis is in bold but the other supporting/associated ones are in italics. However, this format has not been followed for context hypothesis within selection hypothesis on page 8. For better presentation, please follow a uniform format for all four hypotheses.**

We have removed italics within these paragraphs.

9. **Page 9: The escape from stress theory sentence is very lengthy. Consider breaking it into two so that the meaning is effectively delivered.**

We have split the sentence into two: *“The escape from stress theory [2,31,32] also supports the instability hypothesis. However, it suggests that because of the stress of union disruption, the strains of solo parenting, and the reduced financial means, single parents spend less time with their children, are less emotionally supportive, and report more conflict with their children than continuously married parents [31].”*

10. **Page 11: The example may be placed in the section above (versus ending the paragraph with it). “Thus, we expect that economic and/or relationship variables will explain at least some of the differences in between living arrangements” may be used as a concluding statement.**

We have moved the example above the last sentence: *“For example, a wealthier single parent may have more resources than lower income SPC families and maintaining parental contact with the non-resident parent even in LPC arrangements may mitigate the potential negative impact of LPC. Thus, we expect that economic and/or relationship variables will explain at least some of the differences between living arrangements.”*

11. **Page 12: Please provide some elaboration on the PICO components with reference to your study.**

We have included a definition of the PICO components: *“The inclusion and exclusion were based on the PICO (Patient/Problem, Intervention, Comparison, and Outcome) components.”*

12. **Page 13: It has been mentioned that the following population and contexts were included: Western European countries, North American countries, Australia, and New Zealand where divorce and shared physical custody have become increasingly common. The authors are suggested to be support this with existing research.**

We already include this research in the introduction so have not repeated it in the methods section.

13. **Page 15: Under the heading- Comparison of the Empirical Literature with the Theoretical Hypotheses- Most studies supported the fewer resources hypothesis (51.3%) suggesting that children from divorced or separated families have fewer resources than nuclear families with children living in LPC arrangements having the least access to relational and economic resources- is not clear. What are nuclear families with children living in LPC arrangements? How is it a nuclear family if it is an LPC arrangement? – please clarify.**

We can see how this sentence was confusing and have split the sentence into two, which now reads: *“Most studies supported the fewer resources hypothesis (51.3%) suggesting that children from divorced or separated families have fewer resources than nuclear families. Children living in LPC arrangements have the least access to relational and economic resources.”*

14. **Page 17: Methodologies – the heading not clear. What is the justification behind placing it after the Results and before the Discussion.**

It is quite common for systematic reviews to address methodological considerations within the results section which is why we placed this subsection within results rather than discussion. We have changed the name of the subheading to “methodological findings” to make it clear that we are still talking about results.

15. **Page 20: 2^nd^ paragraph: The sentence starts with “Some theories and research findings suggest that the introduction of stepparents…” - and studies are cited in parentheses but it concludes with – but this was not supported by literature. This first part of the sentence mentions certain studies specifically; then how do the authors claim that literature does not support the statement? Are there contradicting studies in the area? If so, then please state this categorically.**

We have changed the wording from “literature” to “present systematic review” to clarify that the conclusion is based on the review.

16. **Page 31: Capitalise R in roles as the word appears after colon in the following reference-**

***Dissing, A. S., Dich, N., Andersen, A. N., Lund, R., & Rod, N. H. (2017). Parental break-ups and stress: roles of age & family structure in 44 509 pre-adolescent children. European Journal of Public Health, 27(5), 829–834. https://doi.org/10.1093/eurpub/ckx040**

We have capitalized the word.
